# Pharmaceutical Co-Crystallization: Regulatory Aspects, Design, Characterization, and Applications

**DOI:** 10.34172/apb.2020.024

**Published:** 2020-02-18

**Authors:** Abdul Raheem Thayyil, Thimmasetty Juturu, Shashank Nayak, Shwetha Kamath

**Affiliations:** Faculty of Industrial Pharmacy, Bapuji Pharmacy College, SS layout, Shamnur road, Davanagere-577004, Karnataka, India. Introduction

**Keywords:** Co-crystals, Evaluation of co-crystals, Hansen solubility parameter, Liquid assisted grinding, Preparation of co-crystals, Solvent evaporation technique

## Abstract

Pharmaceutical co-crystals are novel class of pharmaceutical substances, which possess an apparent probability of advancement of polished physical properties offering stable and patentable solid forms. These multi-component crystalline forms influence pertinent physicochemical parameters like solubility, dissolution rate, chemical stability, physical stability, etc. which in turn result in the materials with superior properties to those of the free drug. Co-crystallization is a process by which the molecular interactions can be altered to optimize the drug properties. Co-crystals comprise a multicomponent system of active pharmaceutical ingredient (API) with a stoichiometric amount of a pharmaceutically acceptable coformer incorporated in the crystal lattice. By manufacturing pharmaceutical co-crystals, the physicochemical properties of a drug can be improved thus multicomponent crystalline materials have received renewed interest in the current scenario due to the easy administration in the pharmaceutical industry. There is an immense amount of literature available on co-crystals. However, there is a lack of an exhaustive review on a selection of coformers and regulations on co-crystals. The review has made an attempt to bridge this gap. The review also describes the methods used to prepare co-crystals with their characterization. Brief description on the pharmaceutical applications of co-crystals has also been incorporated here. Efforts are made to include reported works on co-crystals, which further help to understand the concept of co-crystals in depth.

## Introduction

A multi-component crystalline system is not new but recently the term co-crystal has gained momentum in the glossary of the pharmaceutical world. The physicochemical properties of a drug like melting point, hygroscopicity, solubility, dissolution, and stability are improved for better drug bioavailability, which leads to gain better therapeutic effect of the drug with reduced adverse effects. Solubility as well as dissolution is some of the mechanical characteristics of an active pharmaceutical ingredient (API) that should be thoroughly studied in pharmaceutical drug development.^1-3^

Pharmaceutical co-crystallization is a novel technique that can be employed to get alternatives of salts, solvates, and polymorphs for the modification of API during the process of dosage form design.^4,5^ Making pharmaceutical co-crystals allows the modifications to be made to a crystalline form of an API, which in turn results in an API with altered physicochemical properties without compromising its intended biological properties.^6,7^ The co-crystals can be dissociated into its components by dilution of the co-crystal solution while recent studies also revealed the possibility of co-crystal dissociation by pH-dependent mechanism.^8^

The co-crystals are held, by supramolecular heterosynthons that occur between the functional groups like carboxylic acid–aromatic nitrogen, carboxylic acid–amide and alcohol–pyridine, with non-covalent forces, often including hydrogen bonding.^9,10^ Other forces involved in co-crystallization are ionic and Van der Waals forces, lipophilic-lipophilic interactions and pi-pi interactions.^11^ The co-crystals are different from that of salts due to less extent of proton transfer between drug and coformer. Salt is formed by transferring the proton when the *p* K*a* difference between the partners is sufficiently large.^12^

Pharmaceutical co-crystals could be prepared by different methods like solvent evaporation, anti-solvent addition, crystallization from the melt, solid state grinding, etc.^13-16^ Eddleston et al have used freeze-drying as an approach for the formulation of novel multicomponent crystal forms.^17^

There are limited reports on patents of co-crystals but are expected to grow due to the tremendous improvement in the regulations of co-crystals made by various regulatory authorities across the world.^18^ United States Food and Drug Administration (USFDA) and European Medicine Agency (EMA) are the current two regulatory agencies that regulate the approaches for controlling the quality of pharmaceutical co-crystals. USFDA defined co-crystals as “*Crystalline materials composed of two or more molecules within the same crystal lattice”.*^19^ According to the FDA, co-crystals are taken into account as a drug product intermediate (DPI) that supposes to strengthen the efficiency of an API. EMA defined co-crystal as *“Homogenous crystalline structures made up of two or more components in a definite stoichiometric ratio where the arrangement in the crystal lattice is not based on ionic bonds*”.^20^ The regulations might impact the traits of pharmaceutical co-crystals and their formulations.^6^

## Selection ofcoformers

Coformer with its drug compatibility should be studied prior to its pharmaceutical co-crystal development and are the main challenges to be solved. Coformer screening is the tool used for selecting the coformer that can be formulated as a co-crystal with the drug. The superior candidate is then studied for its physicochemical and pharmacological properties prior to its development to a suitable dosage form. Usually, the coformers are selected from the substances which are approved as generally recognized as safe (GRAS) list by USFDA, these coformers do not affect the pharmacological activities of an API.^21^

### PKabased model

Proton transfer is the phenomenon that occurs in the case of salts. The equation involved in the prediction of co-crystal formation is Δp*K* a=[p*K* a(base) - p*K* a(acid)].^22^ The transfer of proton can be seen if the difference in the p*K* a value is more than 3. If the Δp*K* a value is less than zero, then co-crystal might be formed and the higher value that is more than 3 results in the formation of salts. If the Δp*K* a is in between 0-3, then either co-crystal or salt can be expected.^23^ For example, succinic acid (p*K* a 4.2) forms co-crystal with urea base (p*K* a 0.1) while the salt is formed by using L-lysine base (pKa 9.5).^24^

### Cambridge structural database

Cambridge structural database (CSD) can incorporate to assess the intermolecular hydrogen bonding possibility between different molecules.^16^ CSD single crystal x-ray crystallography can be employed for characterizing the crystal structure of a compound. The resolved structure can be saved in CSD and information can be searched, retrieved, and utilized from the database at any time. ‘Atoms’ and ‘powder cell’ are two examples of the software which can be used to visualize the structure by the information obtained from the CSD.^12^

### Hansen solubility parameter (HSP)

The prediction of miscibility of a drug and coformer, co-crystal formation, is possible by using HSP. In the HSP, the group contribution method is commonly used to determine the HSP since it only requires the structure of the compound.^25,26^ Fedors method, Hoy’s method, and Van Krevelen’s method are the common group contribution methods employed in the calculation of HSP.^27,28^ The theoretical prediction or possibility of the co-crystal formulation is suggested by the scientists Krevelen and Greenhalgh. According to Krevelen, if the deviation in the solubility parameter value of the partners is ≤ 5MPa^1/2^, then co-crystals might be formed. Greenhalgh suggests the formation of co-crystals if the difference is ≤ 7 MPa^1/2^.^22,29^ In addition to this, Salem et al have recently contributed cut-off value 8.18 MPa^1/2^, which is more dependable due to the relaxation of the cut-off value compared to the previous values.^30^

### Hydrogen bonding

From the various studies, it is found that the hydrogen bond donors and acceptors of the partners shall make hydrogen bond. Moreover, the best hydrogen bond donors and acceptors interact within the crystal structure cause to the development of co-crystals.^31^ The formation of hydrogen bonding can be confirmed by FTIR spectroscopy.

### Supramolecular Synthon Approach

**Figure 1:**
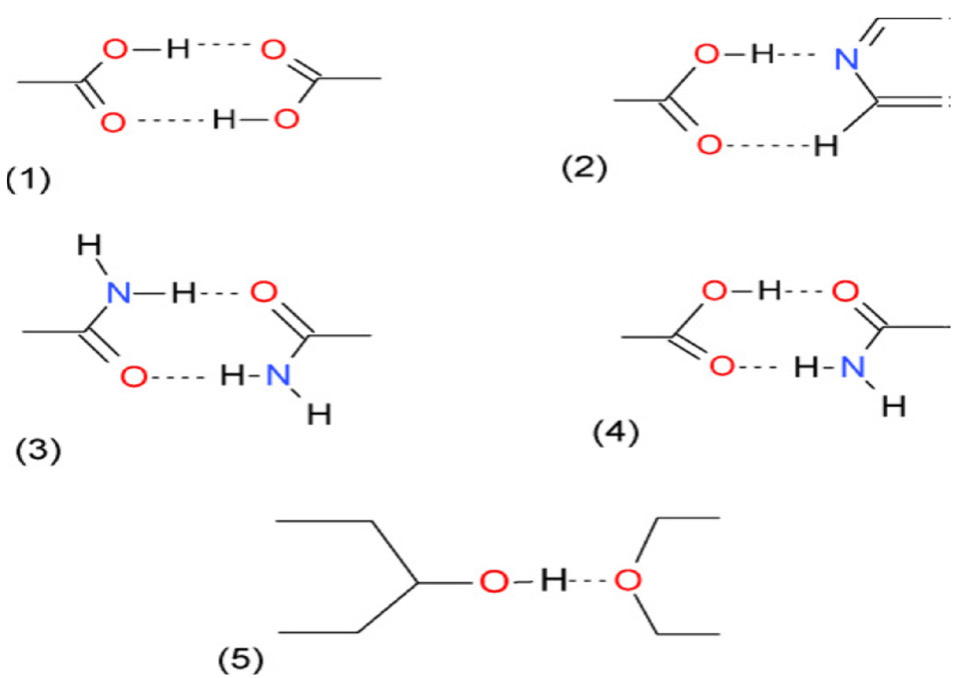
Common supra molecular synthons in crystal engineering: (1) Carboxylic acid diamer (Homosynthon), (2) Carboxylic acid-pyridine (Heterosynthon), (3) Amide diamer (Homosynthon), (4) Carboxylic acid-amide (Heterosynthon), (5) Alcohol-ether (Heterosynthon).^34^

Bolla and Nangia have used the supramolecular synthon approach for screening the coformers for a sulfa drug; acetazolamide.^32^ Supramolecular synthons are further divided into two groups namely supramolecular homosynthons and supramolecular heterosynthons. The former are identical functional groups like two carboxylic acid groups whereas the later consist of different functional groups like carboxylic acid and amide group.^33^ The most commonly used supramolecular synthons are shown in Figure 1.

## Binary and ternary phase diagrams

These phase diagrams illustrate the solubility of either API-coformer (Binary) or API-coformer-solvent (Ternary). DSC analysis can be employed for the construction of binary phase diagram. A ‘W’ shaped diagram will obtain in case of cocrystal formation rather than a ‘V’ shaped diagram, which is found when eutectic mixture is formed between the API and coformer.^35^ Yamashita et al carried out the coformer screening of salts and co-crystals based on binary phase diagram.^36^ Ternary phase diagram (TPD) is a solute-solute-solvent triangular phase diagram that is used for coformer screening in the solution co-crystallization.^37^ Hong et al have used TPD for the successful preparation of myricetin co-crystals.^38^

## Conductor-like screening model for real solvents (CSMO-RS)

CSMO-RS is a computational screening technique where the enthalpy of the API and coformer are calculated and can be implemented for coformer ranking. The excess enthalpy of the API-coformer complex than that of the pure components reveals the possibility of cocrystal formation.^35^ Abramov et al have successfully implemented CSMO-RS theory for the screening of coformers using COSMOthermsoftware.^39^

## Regulation of pharmaceutical co-crystals

The regulation of pharmaceutical co-crystals and their formulations may affect the development and quality control strategies. According to the USFDA, co-crystals are defined as “*Solids which are crystalline materials composed of two or more molecules in the same crystal lattice*”.^23,40^ USFDA was the first agency to give guidance on the regulatory classification of pharmaceutical co-crystals. Co-crystals are classified as DPIs which are expected to improve the physicochemical properties of a drug.^41^ The extent of proton transfer should be proved in the substances claimed as cocrystals. Cocrystals should undergo dissociation to regenerate the drug prior to its location for the therapeutic activity. The guidance gives the data required for the submission as well as its implications of the classification for the new drug applications and abbreviated new drug applications. The recommendations of USFDA do not apply to the already existing materials like complexes, polymers, salts or other non-crystalline forms. The guidance applies only to those materials which have not been determined previously like the pharmaceutical co-crystals.

Recently, the co-crystals are included in the category of ‘new active substances’^24^ and are defined as *“The different salts, esters, ethers, isomers, mixtures of isomers, complexes or derivatives of an active substance shall be considered to be the same active substance unless they differ significantly in properties with regard to safety and/or efficacy”.* From a scientific point of view, solvates including hydrates can be considered as a subgroup of co-crystals however the regulatory context may sometimes differ. Multiple phase materials which are obtained by co-precipitation or physical mixing are not considered as co-crystals by EMA.^19^ According to EMA, the procedure adopted for documentation of co-crystal and salts can be used due to conceptual similarities. In case of any complex coformer usage, additional documentation may be required with the scientific procedure. The comparison of co-crystal parameters of USFDA and EMA based on regulatory status is shown in Table 1.

**Table 1:** Comparison of co-crystal parameters of USFDA and EMA based on regulatory status^19,20,42^

**Co-crystal Parameters**	**USFDA**	**EMA**
Definition	Solids that are crystalline materials composed of two or more molecules in the same crystal lattice.	Homogenous (single phase) crystalline structures made up of two or more components in a definite stoichiometric ratio where the arrangement in the crystal lattice is not based on ionic bonds (as with salts).
Regulatory status	DPI, not regarded as a new API	New Active Substance status depends upon demonstration of efficacy and/or safety
Regulatory regard	Similar to polymorph of the same API	Similar to salts of the same API
Coformers	Neutral guest compound (excipient)	Non-active components/Reagents (excipient)
Chemical interactions	Nonionic	Nonionic
Similarity with API	Yes	Depends upon demonstration of efficacy and/or safety
US-Drug master files (DMF)/EMA-Active substance master file (ASMF)	Not feasible being DPI	Can be filed
Applicable Good manufacturing practice (GMP) regulations/guide	cGMP for drug product	Part II of EU GMP Guide (active substances) and ICH Q7 and in rare cases Part I of EU GMP Guide (finished drug product)

After a thorough look in to the regulatory status, the coformers can be screened based on the method explained in the previous section and the preparation of the cocrystals can be achieved by the techniques described in ‘Preparation of co-crystal section’.

## Preparation of co-crystals

**Figure 2:**
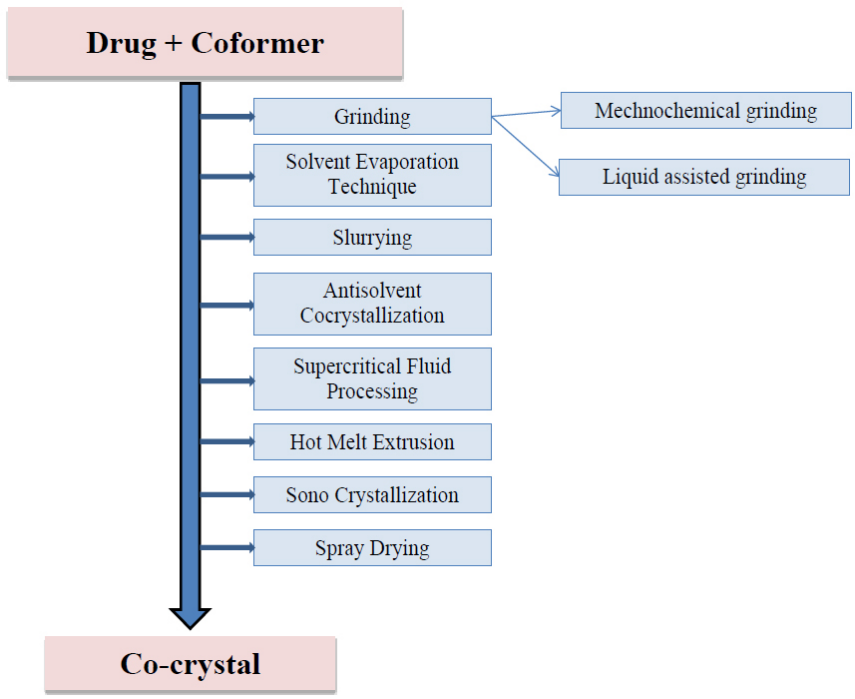
Methods of preparation of co-crystals.

Various methods employed in the preparation of co-crystals are shown in Figure 2.

### Grinding method

#### Mechanochemical grinding

It is also known as solid-state grinding or dry grinding.^9^ The drug and coformers are mixed and crushed either by using mortar and pestle or by mill such as ball mill.^43^ There may be a chance of failure in the formation of co-crystals by this method due to the inability to form crystal arrangement by poor grinding.^12^ The heat produced during the grinding process may affect the stability of the material. Therefore high melting point drugs are usually employed for co-crystallization using mechanochemical grinding. Quashie et al have employed mechanochemical synthesis successfully for the preparation of co-crystals of sulfamethoxazole by using 8-hydroxy-7-iodoquinoline-5-sulfonic acid as a coformer.^44^ Zaini et al have confirmed the formation of sulfamethoxazole-trimethoprim co-crystal by the milling process.^13^

#### Liquid-assisted grinding

Liquid-assisted grinding (LAG) is an alteration of solid-state grinding method with additional small quantity of solvent in the process of development of co-crystal. Here the added solvent acts as a catalyst for the formation of co-crystals.^45^ This method is advantageous when compared to the solvent evaporation technique due to its minimum time consumption and less requirement of the solvent. Thenge et al used LAG for the preparation of diacereinco-crystals from urea and tartaric acid. Both the coformer and drug were taken separately in 1: 1 ratio in a mortar and pestle and ground for 90 minutes by adding a few drops of ethanol approximately 10% of the total weight.^46^

### Solvent evaporation technique

Solvent evaporation is the most common and reliable method used in the preparation of co-crystals. In this technique, the drug and coformer are selected in a proper ratio and dissolved in a common solvent. The solvent is then evaporated at room temperature to get co-crystals. The solubility of drugs and coformer play a great role in the selection of a common solvent. The functional group of drug and coformer undergo intermolecular interaction such as H-bonding and form co-crystal. The requirement of the more quantity of solvent compared to LAG is the main drawback of this method.^12^ Mounika et al prepared fexofenadine-tartaric acid co-crystals by various techniques and concluded that among various methods, the solvent evaporation technique is simple and there is an improvement in the stability and solubility of the drug.^47^ Multidrug co-crystals like sulfadimidine–aspirin can also be prepared by this technique.^48^ Savjani and Pathak prepared acyclovir co-crystals by solvent evaporation, wet grinding, and antisolvent addition. They found that the formation of co-crystals by the solvent evaporation method is better than the other methods used.^10^

### Slurrying

In the slurry co-crystallization, a solvent is added to the API along with its suitable coformer. The solvent is selected according to the solubility of the API and coformer in the solvent. Addition of the coformer to the solution followed by stirring is done for the co-crystals formation.^12^ The solvent is then evaporated at room temperature to obtain co-crystals and can be subjected to PXRD for its characterization.^14,49-51^

### Antisolvent crystallization

Antisolvent crystallization is another method to prepare high-quality co-crystals.^52^ During this process, a second liquid such as organic solvent or buffer is inserted to the drug coformer medium in order to achieve supersaturation. The added liquid should be miscible with solvent so as to precipitate the co-crystal.^9^ The drawback of this method is requirement of large volume of solvent for the preparation.^16^

### Supercritical fluid processing

CO_2_ is the most used supercritical fluid in the preparation of co-crystals due to its penetration ability through the solids. The drug and coformer are added to a stainless steel vessel by dissolving in CO_2_ and rapid expansion of the CO_2_ by gradually decreasing the pressure leads to the formation of co-crystals.^40^ The limited solubility of the drug and coformer in the supercritical fluid and less purity of the co-crystals are the main disadvantages of this method.^9^

### Hot melt extrusion

Hot melt extrusion can be employed for the preparation of co-crystals as a continuous manufacturing process in a single step.^53^ In this method, the co-crystals are prepared by providing heat energy with high intense mixing to the drug and coformer to become miscible in the molten stage.^22^ The criteria for this method are the drug and coformer, which should be miscible at the molten stage.^16^

### Sonocrystallization

Sonoreactor can be used for the preparation of co-crystals. In this method, drugs and coformer are dissolved in a common solvent and kept for sonication at a constant temperature.^54^ Cold water is provided in order to maintain a constant temperature of sonicator.^22^ The air bubbles or voids are produced due to the high energy, that makes size reduction and supersaturation, causes crystallization.^40^ Further evaporation of the solvent accelerates the formation co-crystals.

### Spray drying

Spray dryers can be used for preparing the co-crystals. The partners are dissolved in a common evaporating solvent and sprayed to a hot stream of air for evaporation of the solvent to yield good co-crystals.^22,54^

Once after the preparation of the co-crystals, a rigorous scrutiny has been carried out for the confirmation and purity of the prepared cocrystals. The following section covers some of the characterization techniques that can be adopted for the assessment of co-crystals.

## Characterization of co-crystals

**Figure 3:**
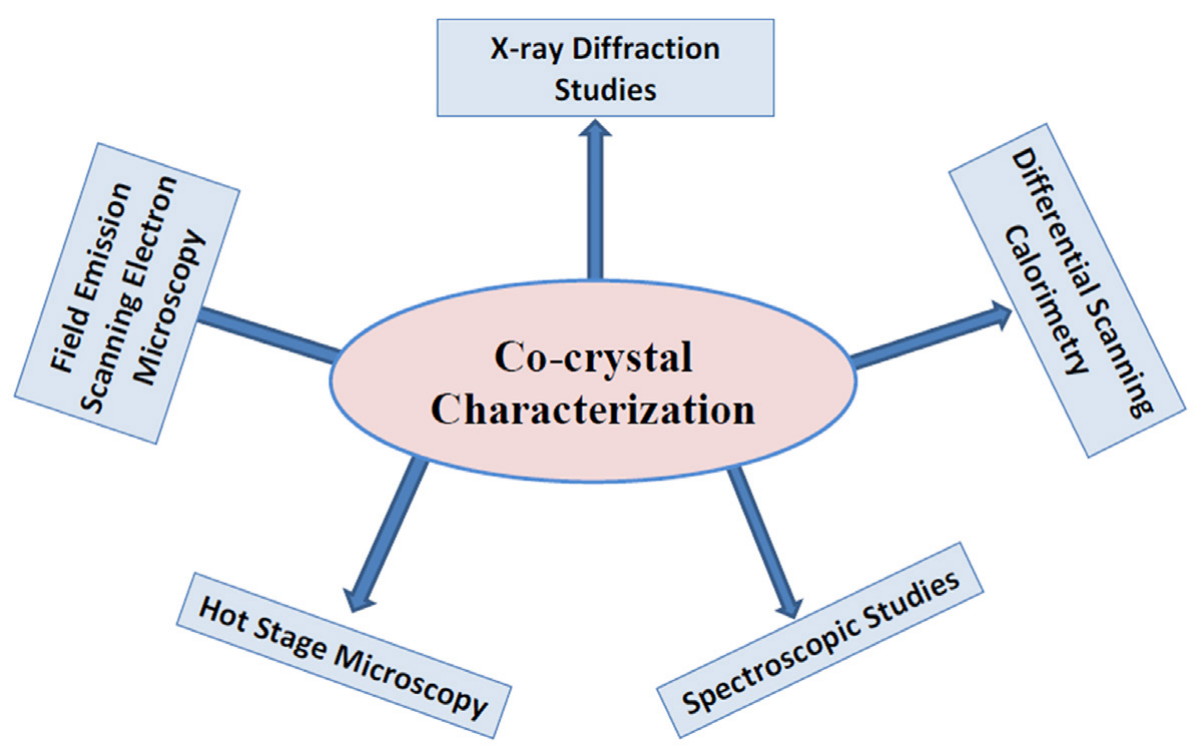
Methods for characterization of co-crystals.

The different instrumental analytical techniques used in the characterization of the cocrystals are shown in Figure 3.

### X-ray diffraction (XRD) studies - single crystalline and powder XRD

This analytical tool is employed for phase identification of unit cells associated with the co-crystal. Complete structural information of co-crystals can be possible to derive from single and powder X-ray crystallography.^55^ Powder XRD is regularly utilized in the identification of various co-crystals by detecting changes in the crystal lattice since different characteristic peaks are associated with different co-crystals while single crystal XRD is mainly employed for structural recognition by using software such as ‘DIFFRAC.SUITE TOPAS’. Difficulty in the procurement of a single crystal is the main problem associated with single crystal XRD.^22^ For powder XRD, the sample is triturated to get a homogenous fine powder. The sample should obey Bragg’s law (*n* λ=2*d* sin θ) for analysis.^14,56^

### Differential scanning calorimetry

Differential scanning calorimetry (DSC) is widely used for the characterization of co-crystals in the pharmaceutical field. In this technique, the co-crystal and pure components are heated at a controlled rate and the obtained thermogram is scrutinized for checking the possibility of co-crystal formation.^9^ In this process, eutectic melt generated at slow heating rates recrystallizes to the co-crystal form and then melts, irrespective of the ratio of drug and coformer.^57^

The thermogram obtained by the DSC scan is used for screening of co-crystals as it allows the co-crystal detection. The thermogram of co-crystals shows a different exothermic peak from that of the pure thermogram of drug and coformer followed by an endothermic peak. The melting point and heat of fusion detected for co-crystals will be different from that of the pure components. If a physical mixture that cannot form co-crystals is heated, then only a single endothermic peak associated with eutectic melting can be seen in thermogram.^40,58^

### Spectroscopy – vibrational, nuclear magnetic resonance

In vibrational spectroscopy (infrared and Raman), the energy absorbed or scattered by the chemical bonds in the co-crystals will be different from that of the pure components, leads to the identification of the structural behavior of co-crystals. In infrared spectroscopy, co-crystals show a different spectrum of bands from the pure drug and coformer due to the formation of hydrogen bonding between them. A clear difference is seen in the bands of functional groups which have undergone hydrogen bonding. For example, a neutral carboxylic acid (COOH) group shows a stronger and weaker tension band of C=O at about 1700 cm^-1^ and 1200 cm^-1^, respectively, and carboxylic anion (COO-) shows a weak tension band in between 1000-1400 cm^-1^ due to the formation of salts. If the H-bonding occurs between OH^….^N, then two broad zones will be witnessed at about 2450 cm^-1^ and 1950 cm^-1^.^40^

Solid-state nuclear magnetic resonanceis widely used for characterization of pharmaceutical co-crystals due to their ability to provide structural information of co-crystals.^59^ This method is also used to distinguish co-crystals and salts since it can detect the degree of proton transfer.^22^ One of the main disadvantages of this method is the low sensitivity of the instrument.^60^

### Field emission scanning electron microscopy (FESEM)

FESEM or topography is used to study the surface morphology of co-crystals. Micrographs of components and co-crystals obtained in the FESEM studies are utilized for the comparison. In the field emission electron microscope, heat energy is not used so-called “cold” source is employed. A strong electric field is utilized to emit the electrons from the surface of the conductor. A tungsten filament with a thin and sharp needle (tip diameter 10–100 nm) is employed as a cathode. The field emission source is attached with a scanning electron microscope for the capture of micrographs of co-crystals.^12,61-63^

### Hot Stage Microscopy

A combination of microscopy and thermal analysis is included in the hot stage microscopystudy. The physicochemical characteristic of a solid form is studied as a function of temperature and time. The changes occurred while heating the co-crystal sample placed on a glass slide are clearly observed under the microscope for assessing the changes such as melting point, melting range, and crystalline transformation.^40^

## Applications of co-crystals in pharmaceutical industry

When compared to other solid-state manipulation methods of a drug such as complexation, solid dispersion, micellar solubilization, cosolvency, etc, co-crystals gained tremendous advantages due to their ease of preparation in the pharmaceutical field.^64,65^ As per the guidelines of USFDA and EMA, all the co-crystals prepared by various coformers can be patented separately, though still there are unclearness lasts. In the recent years, a tremendous increase in the patent application of cocrystals has been found in WIPO patent database.^37^ Four marketed products of co-crystals have been noticed during 2014 to 2017.^66^ The co-crystallization technique can be used for those drugs which are weakly ionized in nature.^22^ Moreover, co-crystals can act as crystallization inhibitor and thereby super saturation can be maintained for a long time during dissolution, which in turn helps to achieve better bioavailability and controlled release of the drug.^2,37,67^ Further the bioavailability of API in cocrystal form can be enhanced by preparing nanosized co-crystals.^34^ But the preparation of nano co-crystals are challenging due to their phase instability of co-crystals in aqueous medium.^68^ Recently multi-drug co-crystal (MDC) is also gaining attraction among pharmaceutical scientists.^69^ MDC have synergistic effects, increased solubility, bioavailability, and potential to stabilize unstable components via intermolecular interactions.^48^ Co-crystals also used for the in process separation and purification of the API.^37^ Rajput et al investigated on various new solid forms of etravirine (anti-HIV drug) and found improved solubility and stability of etravirine co-crystals when compared to salts. It was concluded that the co-crystals approach is a better option for improving the solubility of API compared to salt formation.^70^ Nutraceuticals, which are having good health benefits can also be used as coformers for better-combined health benefits along with the API.^69,71^ By using the coformers such as saccharin sodium, the bitter taste of the API can be modified thereby co-crystallization technique can be utilized in case of fast dissolving tablets. Though there are plenty of co-crystals available in the literature, some of the reported cocrystals are presented, based on the method of preparation, in Table 2.

**Table 2:** Some reported co-crystals with their coformers and method of preparation

**Drug**	**Coformer**	**Method of preparation**
Acyclovir^10^	Tartaric acid, malonic acid, adipic acid	Solvent evaporation
Aripiprazole^72^	Orcinol	
Fexofenadine^47^	Tartaric acid	
Ibuprofen^73,74^	Nicotinamide	
Indomethacin^75,76^	Saccharin	
Itraconazole^77^	Succinic acid	
Myricetin^78-81^	Isonicotinamide, caffeine, proline, nicotinamide, 4-cyano pyridine	
Valsartan^82^	Succinic acid	
Aceclofenac^83^	Nicotinamide	Neat grinding
Aripiprazole^72^	Orcinol	
Etodolac^84^	Salicylic acid, benzoic acid, malonic acid, cinnamic acid, tartaric acid, PABA, hippuric acid, ferulic acid, maleic acid, glutaric acid	
Acemetacin^85^	Isonicotinamide, picolinamide, caprolactam	Cooling crystallization
Chloral hydrate^86^	Betain	
Darunavir^87^	Succinic acid	
Atorvastatin calcium^88^	Aspartame	Slurry method
Baicalein^89^	Nicotinamide	High-pressure homogenization
Caffeine^90-92^	Oxalic acid, glutaric acid	Twin screw extrusion, spray congealing, cooling crystallization
Carbamazepine^90^	Saccharin	Twin screw extrusion
Nicotinamide^90^	Trance Cinnamic acid	
Furosemide^91^	Anthranilamide, 4-toluamide, 2-picolinic acid, isoniazid, theophylline, 2,3,5,6-tetramethyl pyrazine, 2-picolinamide, pyrazine, piperazine	Liquid-assisted grinding
Gliclazide^92^	Sebacic acid, α-hydroxyacetic acid	
Entacapone^93^	Theophylline, nicotinamide, Acetamide isonicotinamide, pyrazinamide, isoniazid	
Salicylic acid^94^	Nicotinic acid, DL- phenylalanine, 6-hydroxy nicotinic acid, benzamide, isonicotinamide	
Piroxicam^95^	Sodium acetate, saccharin sodium, urea, nicotinamide, resorcinol	Dry grinding
Diacerin^46^	Urea, tartaric acid	Solvent drop grinding
Fenofibrate^96^	Nicotinamide	
Quercetin^97^	Caffeine, nicotinamide	Electrospray technique
Exemestane^98^	Maleic acid	Slurry crystallization
Isoniazide^99^	Vanillic acid, ferulic acid, caffeic acid, resorcinol	
Megestrol acetate^98^	Saccharin	
P-coumaric acid^100^	Nicotinamide	
Mefenamic acid^101^	Nicotinamide	Co-milling

## Conclusion

Pharmaceutical co-crystals are a novel class of pharmaceutical substances which are proposing an apparent probability of advancement of polished physical properties offering new stable and patentable solid forms. These multi-component crystalline forms influence pertinent physicochemical parameters like solubility, dissolution rate, chemical stability, physical stability, etc. which in turn results in the materials with superior properties to those of the free drug. This review offers an insight to regulatory prospects with a standard description of various methods that can be employed in the preparation of co-crystals followed by their characterization. Prior to the preparation of co-crystals, it is advisable to perform the screening of the coformers and prediction of co-crystal formation to avoid unexpected results. As of our understanding, the intermolecular interaction that defines the formation of co-crystals could become a strengthening tool for assisting co-crystal based dosage form design. It is expected that, a drastic adaptation of the co-crystallization technique to prepare pharmaceutically useful co-crystals in the coming years with well elaborated regulatory frame work.

## Ethical Issues

Not applicable

## Conflict of Interest

The authors declare no conflict of interest.

## Acknowledgments

The authors are thankful to Advance Research Wing, Rajiv Gandhi University of Health Sciences, Karnataka, for the financial support and Bapuji Pharmacy College, Davangere for providing all the library facilities.
